# Mitomycin C in Ahmed Glaucoma Valve Implant Affects Surgical Outcomes

**DOI:** 10.3390/bioengineering12080859

**Published:** 2025-08-10

**Authors:** Wei-Chun Lin, Sen Yang, Michelle R. Hribar, Aiyin Chen

**Affiliations:** Casey Eye Institute, Department of Ophthalmology, Oregon Health & Science University, 3181 S.W. Sam Jackson Park Rd, Portland, OR 97239, USA

**Keywords:** drug discovery, outcome research, glaucoma, surgery, mitomycin C, wound healing

## Abstract

Glaucoma is the leading cause of irreversible blindness worldwide, and the Ahmed Glaucoma Valve (AGV) implant is one of the most commonly performed surgeries to prevent glaucoma-related visual impairment. Mitomycin C is an anti-fibrotic agent that may prevent failure of AGV. This is a retrospective case–control study to evaluate surgical outcomes for patients undergoing AGV with adjunct mitomycin C (MMC) injections compared to those without MMC. Among the 142 eyes, 50 received adjunct MMC compared to 92 without MMC injections. IOPs at post-operative months 1, 3, and 6 were significantly lower in the MMC eyes (9.40, 12.01, 12.63 mmHg) compared to the No-MMC eyes (16.86, 15.87, 15.65 mmHg; *p* < 0.01). The number of post-operative glaucoma medications for the MMC group was lower at 1, 3, and 6 months (0.3, 0.4, 0.59) compared to the No-MMC group (0.7, 0.97, 1.05; *p* < 0.05). The difference in IOP and the number of medications was not statistically significant by 12 months. Adjunct MMC was associated with more transient hypotony but no long-term complications. These findings suggest that adjunct MMC improves short-term but not long-term surgical outcomes in AGV glaucoma implants.

## 1. Introduction

Glaucoma continues to be the leading cause of irreversible blindness and is projected to affect over 111 million individuals worldwide by 2040 [1,2]. The primary treatment modality is to lower intraocular pressure (IOP) with ophthalmic medication and surgical intervention. Glaucoma drainage device implantation is one of the most commonly performed glaucoma surgeries [3,4]. Among the drainage devices, the Ahmed Glaucoma Valve (AGV; New World Medical, Rancho Cucamonga, CA, USA) can reduce IOP immediately through a valved mechanism with a lower incidence of post-operative hypotony [5]. However, more than half of AGV implant fails within 5 years, and fibrosis around the implant is the leading reason for device failure [5]. Various anti-inflammatory agents, such as steroids, anti-VEGF, and antifibrotic medications, have been employed to address this issue. However, the success rates of these interventions have varied, indicating a need for ongoing research and optimization of treatment strategies [6].

Antifibrotic agents, including mitomycin C (MMC) and 5-fluorouracil (5-FU), have improved the outcome of glaucoma surgeries like trabeculectomy [7,8], but their utility in glaucoma drainage devices is inconclusive. Antifibrotic agents reduce fibrovascular proliferation and prevent bleb fibrosis [9,10]. Only a small body of studies exists on antifibrotic agents in glaucoma drainage devices, most with small sample sizes [11,12,13,14]. Moreover, many studies were performed on refractory or secondary glaucoma, and the ideal medication dosage, frequency, and application method remain undefined. There remains a knowledge gap in the effectiveness of adjunct MMC in primary AGV surgery.

In this retrospective study, we assessed adjunct MMC’s long-term effects on IOP reduction and the medication burdens in patients who received primary AGV surgery. Specifically, we focused on those who received a single intraoperative injection of MMC (0.04 mg), followed by a prn post-operative MMC injection. By leveraging electronic health record (EMR) data and corroborating it with chart reviews, we aimed to conduct the largest retrospective study on this topic.

## 2. Methods

### 2.1. Patient Selection

This retrospective case–control study examined patients who underwent primary AGV implantation with or without adjunct subconjunctival injections of MMC at the Casey Eye Institute, Oregon Health and Science University (OHSU) between January 2017 and August 2020. This study was approved by the Institutional Review Board at OHSU (IRB Number MODCR00029051). Due to the nature of the EHR study, participants did not give informed consent to participate, as no participants were recruited in the study. Inclusion criteria were adults (18+) undergoing primary AGV implantation (naïve to prior glaucoma surgery) with or without cataract extraction by any of the five glaucoma specialists. We included only one eye per patient, selecting the first operative eye if both eyes received the same surgery. Patients with less than three months of post-operative follow-up were excluded, as well as those who had previous trabeculectomy or underwent concomitant surgeries other than phacoemulsification. Preoperative data were extracted from the EMR and included age at the time of the surgery, sex, race, and glaucoma diagnosis, as well as mean IOP and the number of glaucoma medications up to 6 months before the surgery. Patients and the public were not involved in the design, conduct, reporting, or dissemination plans of our research.

### 2.2. Surgical Procedure and MMC Application

**Figure 1:**
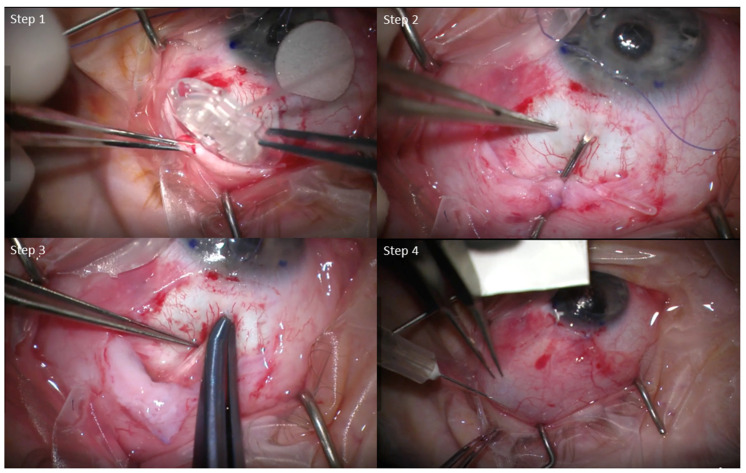
Surgical Steps of Ahmed Valve FP7 Implantation with Mitomycin-C Adjunct. **Step 1**: Ahmed valve FP7 was placed in the subtenon space after conjunctival opening and dissection. **Step 2**: A 23-G needle was used to tunnel into the eye to create a track for tube insertion. **Step 3**: The Tube was inserted into the anterior chamber or sulcus. **Step 4**: After wound closure, mitomycin-C was injected over or adjacent to the plate.

All surgeries were performed in a similar fashion, with minor individual differences between surgeons. Briefly, a limbal conjunctival peritomy and sub-tenon dissection were performed. The AGV FP7 was primed and sutured in place using nylon sutures. The tube was inserted either into the anterior chamber or sulcus. A donor scleral or corneal patch graft was placed at the tube entry site to prevent exposure. The conjunctiva was closed using either nylon or vicryl sutures, and in combination with Tisseal fibrin glue in some cases as necessary. Subconjunctival or intracameral antibiotics were given at the conclusion of the case (Figure 1). The surgeries of the MMC group were performed by a single surgeon (AC), while the No-MMC surgeries were performed by the remaining four surgeons in the institution due to practice patterns.

For the MMC group, all patients received a subconjunctival injection of 0.1 mL of MMC (0.4 mg/mL) over the AGV plate while the eye was retracted as far down as possible at the conclusion of the case. The eye was irrigated with copious balanced salt solution after MMC injection. Eyes in the MMC group were given an additional post-operative injection based on their risk factors (i.e., age, race, or ocular diagnosis such as neovascular glaucoma), target IOP, or increasing IOP during the post-operative course. Post-operative MMC injections were performed within the first two months. Briefly, eyes were treated with topical antibiotics and proparacaine drops. A cotton swab soaked in lidocaine was used to apply local anesthetics to the intended conjunctival area of injection overlying the AGV plate. The same MMC dosage (0.1 mL of 0.4 mg/mL) was injected subconjunctivally, and the eye was rinsed with copious amounts of balanced salt solution. The patient was instructed to use topical antibiotics for 3 days after the injection.

### 2.3. Outcome Measures

The primary outcomes were IOP and glaucoma medication count at 1, 3, 6, and 12 months post-operatively. We obtained the number of glaucoma medications at each visit using the following methods. First, we extracted patients’ glaucoma medication from their EMR medication lists by matching medications to 43 unique medication IDs. We then used a manual chart review of patients’ progress notes to identify and exclude medication errors, such as incomplete records and failed discontinuation. Next, combination medications were re-coded using medication class to exclude duplicate medications. Finally, glaucoma medications were categorized into eight drug classes based on their pharmacologic mechanisms: prostaglandin analogs, beta-blockers, topical and oral carbonic anhydrase inhibitors, alpha-agonists, Rho-kinase inhibitors, nitric oxide donors, and cholinergic agonists. Twenty patient encounters were manually reviewed to cross-validate the accuracy of glaucoma medications. The secondary outcomes include the incidence of hypertensive phase, defined as IOP ≥ 21 mmHg during any post-operative visits between 1 and 3 months. Post-operative complications included hypotony (IOP < 6 mmHg), additional glaucoma procedures, blebitis, and endophthalmitis. All cases with complications were verified by manual chart reviews.

### 2.4. Statistical Analysis

Continuous variables were analyzed using Welch’s *t*-test, and the Mann–Whitney U test assessed non-normally distributed data. Categorical variables were compared using Chi-squared and Fisher’s exact tests. We evaluated the effects of post-operative MMC injections using the Mann–Whitney U test. We compared surgical failure rates using Kaplan–Meier survival analysis and the log-rank test. Surgical failure was defined as: (1) IOP exceeding 21 or 15 mmHg after one month post-operatively, or (2) reduction of IOP ≤ 20% compared to the preoperative value, or (3) the need for reoperation. Statistical significance was set at *p* < 0.05, with analyses performed in Python (3.10.1) and R (4.2.3).

## 3. Results

A total of 183 eyes from 163 patients underwent AGV implantation during the study period. Twenty patients underwent the procedure in both eyes, and only the first eye was included. After excluding 21 eyes due to prior surgeries (18) or incomplete data (3), 142 eyes were analyzed; 50 received intraoperative MMC, and 92 received no MMC injection. The No-MMC group was statistically younger than the MMC group (68.49 ± 13.16 vs. 75.01 ± 10.92, *p* < 0.01), but no other significant demographic differences were observed (Table 1).

**Figure 2:**
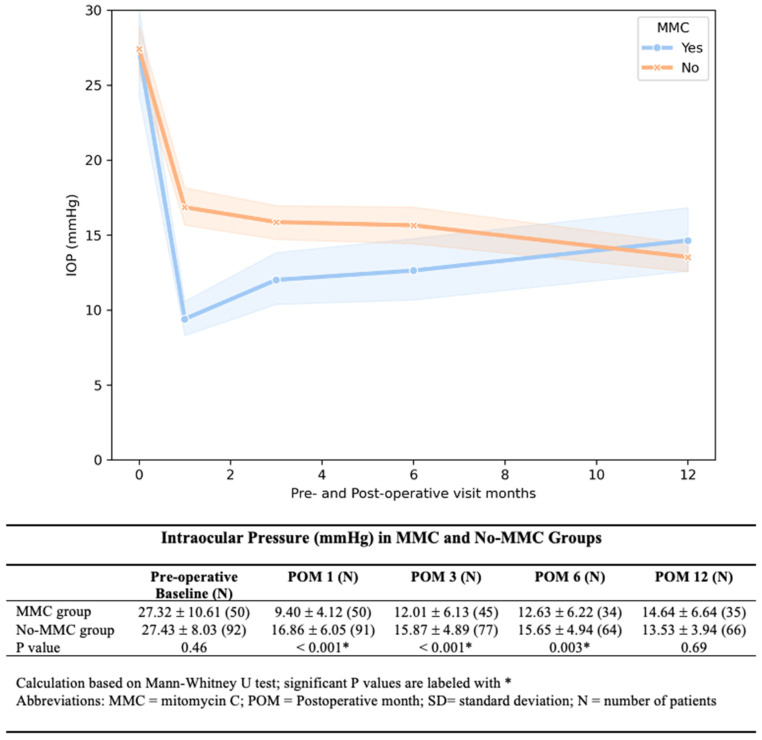
**Intraocular Pressure between the MMC and No-MMC groups.** Post-operative IOP was significantly lower in the MMC group compared to the No-MMC group at 1, 3, and 6 months after AGV implantation.

**Figure 3:**
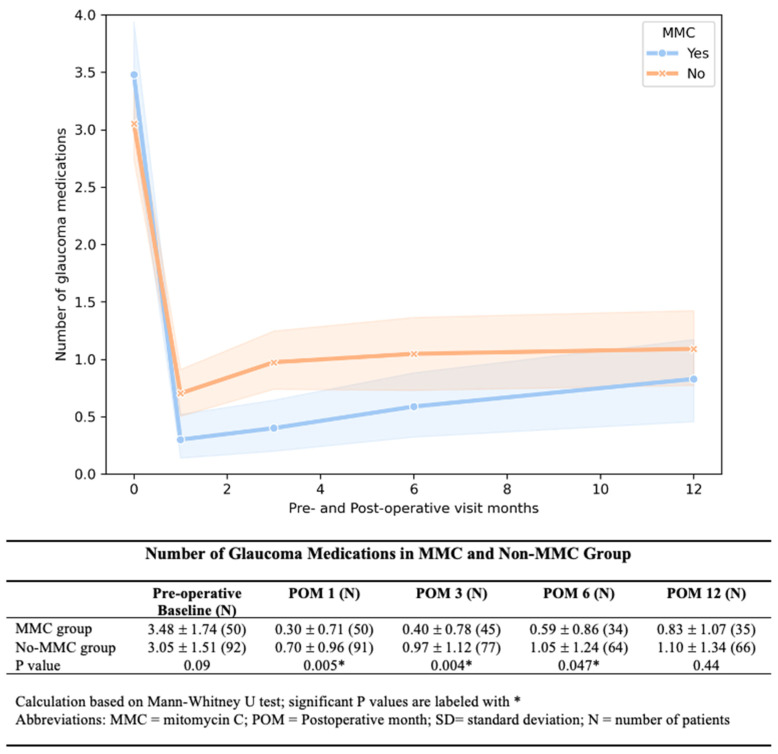
**Number of glaucoma medications between the MMC and No-MMC groups**. The patients in the MMC group needed significantly fewer medications at all post-operative follow-up visits for up to 1 year.

The MMC group showed significantly lower IOP at 1, 3, and 6 months post-operatively. The IOP difference diminished with each time point, and by 12 months, the difference was no longer observed (Figure 2). The MMC group required significantly fewer medications at 1, 3, and 6 months after AGV implantation (Figure 3). We found a lower rate of hypertensive phase in the MMC group (10% vs. 33.69%, *p* < 0.01.). By 12 months, medication-free rates were similar between groups (57% MMC vs. 53% No-MMC, *p* = 0.33).

**Figure 4:**
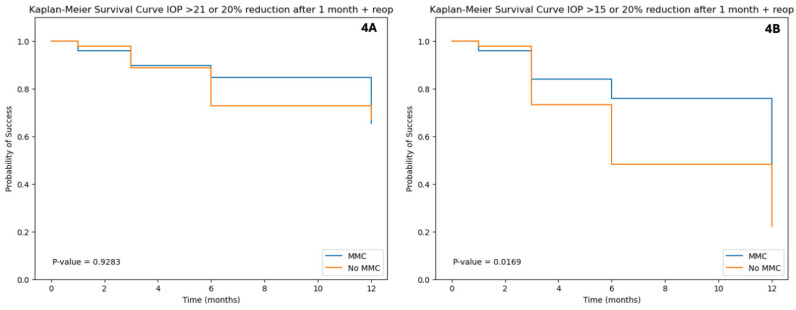
**Kaplan–Meier survival curves comparing surgical failure rates between the MMC and No-MMC groups**. Surgical failure was defined by any of the following criteria: IOP exceeding 21 mmHg (**4A**) or 15 mmHg (**4B**) in any visit after one month post-operatively, an IOP reduction of 20% or less relative to the preoperative value, or reoperation. Abbreviations: MMC = mitomycin C; IOP = intraocular Pressure.

Kaplan–Meier survival analysis was used to compare failure rates between the two groups (Figure 4). Using the above-mentioned criteria, the cumulative probability of success at 12 months using IOP > 21 mmHg as failure criteria was 72% for both groups (*p* = 0.928 by log-rank test). However, using IOP > 15 mmHg as a criterion, the MMC group had a higher cumulative success rate (48%) compared to the No-MMC group (32%, *p* = 0.017; log-rank test).

In the MMC group, 76% received only intraoperative MMC, and 24% received *PRN* injections post-operatively. The injections were performed within 2 months after surgery, with a mean of 22 days. *prn* MMC injections did not significantly impact IOP outcomes (*p* > 0.2 at all time points; Mann–Whitney U test).

The MMC group had a significantly lower number of follow-up visits compared to the No-MMC group during the 90-day global period (Mean 5.73 ± 2.98 vs. 4.02 ± 1.96, *p* = 0.0001). The number of patients lost to follow-up increased with time in both groups. At 12 months post-operatively, 70.0% (35/50) in the MMC and 71.7% (66/92) in the No-MMC group had follow-up data. There was no statistically significant difference between the loss to follow-up rate between the MMC and no-MMC groups at any follow-up time points.

Complications were similar between the two groups, except for hypotony (IOP < 6 mmHg) which was more prevalent in the MMC group (15 patients vs. 6 patients, *p* < 0.01), though it was mostly transient (Table 2). Persistent hypotony occurred in four MMC and two No-MMC eyes, with some cases (3 MMC and 2 No-MMC) accompanied by choroidal detachment. One No-MMC eye experienced suprachoroidal hemorrhage. No significant differences between the groups were noted in reoperation rates (*p* = 0.64) or infection (*p* = 0.28) rates.

## 4. Discussion

**Figure 5:**
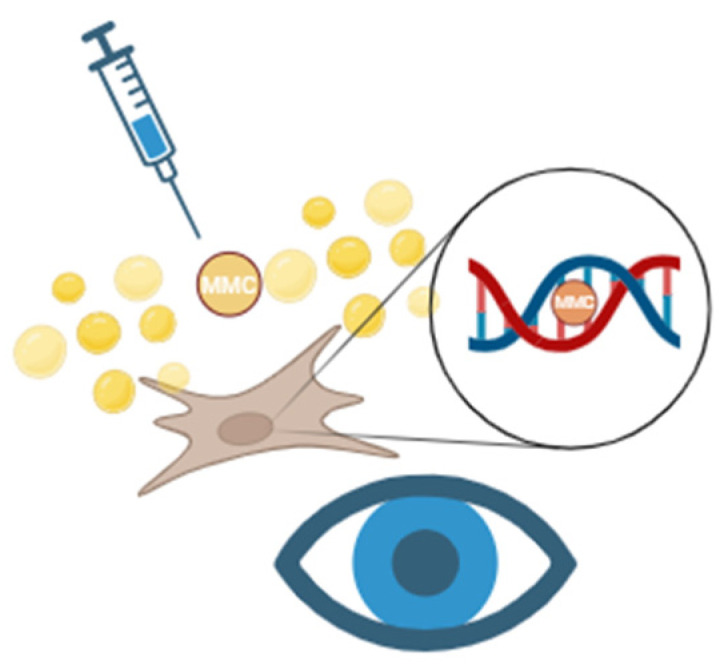
Mitomycin C (MMC) mechanism of action. MMC is injected in the subtenon space and activated within tenon fibroblasts. It causes cross-linking between the DNA double strands and prevents DNA replication or RNA transcription. This action prevents tenon proliferation and fibrosis, thus improving glaucoma surgical outcome. Created in https://BioRender.com.

This study evaluated primary AGV outcomes with adjunct MMC usage and found that a single 0.4 mg/mL MMC injection during surgery had significant effects up to 6 months post-operatively, but this effect did not persist at 12 months. Our study was unique in several ways. First, it was the largest retrospective study on this subject to date, with 142 total eyes. Previous studies included sample sizes ranging between 21 and 75 eyes (mean of 43) with the study summarized in Table 3 [11,12,13,14]. Second, the application of MMC had a higher concentration of 0.04 mg via subconjunctival injections rather than removable soaked sponges [15,16]. Thirdly, the adjunctive MMC was performed as primary surgery rather than refractory glaucoma, and the majority of the patients have primary open angle glaucoma. MMC inhibits fibroblast proliferation by acting as an alkylating agent to prevent DNA replication and transcription, halting the cell cycle of fibroblasts. MMC also creates reactive oxygen radicals, which contribute to apoptosis and cell death [10] (Figure 5).

Our study demonstrated that MMC in AGV implantation was associated with lower post-operative IOP and glaucoma medication for up to 6 months but not at 12 month. The IOP difference diminished over time due to the gradual increase in IOP in the MMC group. For medication usage, both the MMC and No-MMC groups required more glaucoma medicine with time. While the No-MMC group started using an average of 1 glaucoma medication by 3 months, the MMC group did not reach this threshold until 12 months after surgery. The No-MMC eyes used more glaucoma medications than the MMC group at each follow-up time point, but the difference is no longer statistically significant at 12 months. Our study agreed with previous studies that the MMC was insufficient to make a long-term difference. The initial MMC effect diminished with time. Further research is needed to optimize the timing and use of either repeat MMC or steroids to control wound healing and prevent chronic failure.

Survival analysis showed a statistically significant difference for MMC vs. No-MMC groups when one of the failure criteria was set for IOP > 15 mmHg, but there was no difference for failure criteria set at IOP > 21 mmHg. Adjunct MMC may help patients who require a lower IOP target of less than 15 mmHg.

We found that the MMC group had significantly fewer post-operative clinic visits compared to the No-MMC group during the global period of the first 90 days. There are several possible reasons. The No-MMC group had a 3-fold increase in the rate of hypertensive phase compared to the MMC group and, therefore, likely requires more IOP monitoring. There may also be differences in practice patterns between the surgeons. The decreased post-operative visits mean less global resource use (time, productivity, space), which can offset the cost of MMC.

For surgical complications, MMC use contributed to the increased incidence of hypotony. Fortunately, most hypotony was transient. No statistical differences between the groups were found in reoperation or infection rates. However, we noted anecdotally that some MMC eyes had more avascular bleb over the plate, and therefore, it remained to be seen if the MMC carried a long-term risk of infections. Further studies with extended follow-up periods are needed.

There are several limitations of the study. First, the predominantly Caucasian patient base may limit the generalizability of the findings to more racially diverse populations, given the racial differences in glaucoma surgical outcomes [19,20,21]. Second, only one glaucoma surgeon in this study used adjunct MMC with AGV, and therefore, it is not possible to eliminate the surgeon factor as a potential confounder. However, a review of surgical techniques showed only minor differences between surgeons, and the single surgeon in the MMC group inherently minimized variability within the group. Third, we were not able to access the bleb morphology as the etiology for implant failure due to the inconsistent exam documentation among the surgeons. Last, long-term loss to follow-up rate may affect results in a retrospective study, although there was no significant difference between the follow-up rates of the two groups. Consequently, we were not able to extend the study past 12 months due to insufficient patients remaining in the study. A multi-centered randomized clinical trial has been completed using standardized intraoperative MMC, followed by two post-operative injections. Their results will shine light on optimal MMC usage.

In conclusion, primary AGV implantation with 0.4 mg/mL MMC injection was associated with a short-term but not long-term reduction in post-operative IOP and glaucoma medications. Future methods and studies are needed to extend MMC’s short-term effect to improve AGV outcomes.

## Figures and Tables

**Table 1:** Baseline Demographic and Clinical Characteristics.

	No-MMC Cohort (92 Eyes)	MMC Cohort (50 Eyes)	*p* Value
**Demographic**			
Age (years ± SD)	68.49 (13.16)	75.01 (10.92)	0.003 *
Gender (%)			0.06
Male	48 (50%)	17 (39%)	
Female	44 (50%)	33 (61%)	
Race			0.409
White	75 (82%)	46(92%)	
Black	7 (8%)	2 (4%)	
Asian	5 (5%)	1 (2%)	
Others	5 (5%)	1 (2%)	
**Clinical Characteristics**			
Intraocular Pressure (mmHg ± SD)	27.43 (8.03)	27.32 (10.61)	0.46
Number of Glaucoma Medications	3.03 (1.48)	3.41 (1.75)	0.091
**Glaucoma Diagnosis**, POAG	70 (76%)	37 (74%)	1.0
**Glaucoma Diagnosis**, others	22 (24%)	13 (26%)	
PACG	2 (2%)	1 (2%)	
PXEG	10 (11%)	5 (10%)	
Pigmentary glaucoma	4 (4%)	1 (2%)	
Neovascular glaucoma	0 (0%)	1 (2%)	
Secondary glaucoma	2 (2%)	3 (6%)	
Low-tension glaucoma	1 (1%)	1 (2%)	
Congenital glaucoma	2 (2%)	0 (0%)	
Glaucoma due to trauma	0 (0%)	1 (2%)	
Inflammatory	1 (1%)	0 (0%)	

Calculation based on Pearson’s chi-square test (Sex, Race, and Glaucoma Diagnosis), Welch’s *t*-test (Age), and Mann–Whitney U test (Intraocular Pressure and Number of Glaucoma Medications). * *p*-values were two sided and *p* ≤ 0.05 were considered statistically significant. Abbreviations: MMC = mitomycin C; SD = standard deviation; POAG = Primary open angle glaucoma; PACG = Primary angle-closure glaucoma; PXEG = Pseudoexfoliation glaucoma.

**Table 2:** Post-operative Complications in MMC vs. No-MMC Groups.

	MMC 50 Patients	No-MMC 92 Patients	*p*-Value
**Hypotony (IOP < 6 during any post-op visit)**	15 (30%)	6 (5%)	<0.01 *
Transient Hypotony (=1 visit)	11 (73%)	4 (67%)	
Persistent Hypotony (>1 visits)	4 (27%)	2 (33%)	
Vision Decrease > 1 Snellen line	2 (13%)	0 (0%)	
Corneal Striae	4 (27%)	3 (50%)	
Choroidal Detachment	3 (20%)	2 (33%)	
Bleb Leak	1 (7%)	0 (0%)	
Maculopathy	0 (0%)	0 (0%)	
No ocular findings	7 (47%)	2 (33%)	
**Reoperations**	3 (6%)	9 (10%)	0.64
Second shunt	1 (33%)	1 (11%)	
Shunt revision	2 (67%)	3 (33%)	
Endo cyclophotocoagulation	0 (0%)	0 (0%)	
Transscleral cyclophotocoagulation	0 (0%)	5 (56%)	
**Bleb Associated Infections**	2 (4%)	1 (1%)	0.28
Endophthalmitis	1 (50%)	1 (100%)	
Blebitis	1 (50%)	0 (0%)	

Calculation was performed using either the Chi-Squared Test or Fisher’s Exact Test. Two-sided *p*-values were calculated, and statistical significance was determined with a significance level of *p* ≤ 0.05. Abbreviations: MMC = mitomycin C; IOP = intraocular pressure. Significant p values were denoted with *.

**Table 3:** Summary of Prior Studies on Adjunctive Mitomycin C in Glaucoma Surgery.

Author(s)	Year	Country	Result
Perkins T. W. et al. [12]	1995	USA	Retrospective case–control of 41 eyes with a follow-up duration of 12 months. MMC of 0.4 mg/mL applied via soaked sponges for 5 min to Molteno tube shunt. IOP reduced from 34.0 mmHg at baseline to 13.0 mmHg for MMC compared to control from 32 mmHg to 15.7 mmHg (*p* = 0.08).
Lee D. et al. [13]	1997	USA	Retrospective case–control of 95 eyes with a follow-up duration of 21 months. MMC of 0.5 mg/mL applied via soaked sponges for 3–5 min to Molteno tube shunt. IOP reduced from 35.1 mmHg at baseline to 18.0 mmHg for MMC compared to control from 36.5 mmHg to 17.3 mmHg (*p* < 0.0001).
Cantor L. et al. [14]	1998	USA	Randomized controlled trial of 40 eyes with a follow-up duration of 12 months. MMC of 0.4 mg/mL applied via soaked sponges for 2 min to Molteno tube shunt. IOP reduced from 41.5 mmHg at baseline to 15.6 mmHg for MMC compared to control from 34.7 mmHg to 15.3 mmHg (*p* > 0.05).
Costa et al. [17]	2004	United Kingdom	Randomized controlled trial of 60 eyes with a follow-up duration of 12.3 months. MMC of 0.5 mg/mL applied via soaked sponges for 5 min to Ahmed tube shunt. IOP reduced from 31.6 mmHg at baseline to 15.1 mmHg for MMC compared to control from 35.4 mmHg to 14.3 mmHg (*p* = 0.864).
Kurnaz et al. [18]	2005	Turkey	Prospective case–control of 48 eyes with a follow-up duration of 12 months. MMC of 0.5 mg/mL applied via soaked sponges for 3 min to Ahmed tube shunt. IOP reduced from 39.6 mmHg at baseline to 17.6 mmHg for MMC compared to control from 42.3 mmHg to 19.2 mmHg (*p* = 0.03).
Yazdani S. et al. [11]	2016	Iran	Randomized controlled trial of 68 eyes with a follow-up duration of 12 months. MMC of 0.2 mg/mL applied via soaked Q-tips for 3 min to Ahmed tube shunt. IOP reduction was similar in MMC group compared to control group without statistical significance. Exact post-op IOP was not reported in this article.
Perez C. I. et al. [15]	2021	USA	Retrospective comparative study of 37 eyes with a follow-up duration of 6 months. MMC of 0.25 mg/mL injected over the plate intra-op, at week 1 and 5 post-operatively. IOP reduced from 31.8 mmHg at baseline to 14.0 mmHg compared to control from 27.6 mmHg to 14.7 mmHg (*p* = 0.6).
Our study (Lin et al.)	2025	USA	Retrospective case–control of 142 eyes with a follow-up duration of 12 months. MMC of 0.04 mg was applied as subtenon injection to Ahmed tube shunt. IOP reduced from 27.3 mmHg at baseline to 12.6 mmHg for MMC compared to 27.4 mmHg to 15.7 mmHg up to 6 months follow-up (*p* = 0.003).

## Data Availability

Data is available upon request by sending emails to the corresponding author.
